# A diagnostic, monitoring, and predictive tool for patients with complex valvular, vascular and ventricular diseases

**DOI:** 10.1038/s41598-020-63728-8

**Published:** 2020-04-23

**Authors:** Zahra Keshavarz-Motamed

**Affiliations:** 1 Department of Mechanical Engineering, McMaster University, Hamilton, ON Canada; 2 School of Biomedical Engineering, McMaster University, Hamilton, ON Canada; 3 School of Computational Science and Engineering, McMaster University, Hamilton, ON Canada

**Keywords:** Interventional cardiology, Biomedical engineering

## Abstract

Hemodynamics quantification is critically useful for accurate and early diagnosis, but we still lack proper diagnosticmethods for many cardiovascular diseases. Furthermore, as most interventions intend to recover the healthy condition, the ability to monitor and predict hemodynamics following interventions can have significant impacts on saving lives. Predictive methods are rare, enabling prediction of effects of interventions, allowing timely and personalized interventions and helping critical clinical decision making about life-threatening risks based on quantitative data. In this study, an innovative non-invasive imaged-based patient-specific diagnostic, monitoring and predictive tool (called C3VI-CMF) was developed, enabling quantifying (1) details of physiological flow and pressures through the heart and circulatory system; (2) heart function metrics. C3VI-CMF also predicts the breakdown of the effects of each disease constituents on the heart function. Presently, neither of these can be obtained noninvasively in patients and when invasive procedures are undertaken, the collected metrics cannot be by any means as complete as the ones C3VI-CMF provides. C3VI-CMF purposefully uses a limited number of noninvasive input parameters all of which can be measured using Doppler echocardiography and sphygmomanometer. Validation of C3VI-CMF, against cardiac catheterization in forty-nine patients with complex cardiovascular diseases, showed very good agreement with the measurements.

## Introduction

Cardiovascular disease is the leading cause of death globally, taking more lives than all forms of cancer combined and is the leading cause of burden on healthcare around the world as well. It is expected to remain the first cause of death by 2030 in the world^1^. *Complex valvular-vascular-ventricular interactions (C3VI) is the most general and fundamentally challenging condition* *in which multiple valvular, vascular and ventricular pathologies have mechanical interactions with one another wherein physical phenomena associated with each pathology amplify effects of others on the cardiovascular system*^2–6^. Examples of components of C3VI include: valvular disease (e.g., aortic valve stenosis, mitral valve stenosis, aortic valve regurgitation and mitral valve insufficiency), ventricular disease (e.g., left ventricle dysfunction and heart failure), vascular disease (e.g., hypertension), paravalvular leaks, and LV outflow tract obstruction in patients with implanted cardiovascular devices such as transcatheter valve replacement (TVR), changes due to surgical procedures for C3VI (e.g., valve replacement and left ventricular reconstructive surgery) and etc^2,4–7^.

*“Cardiology is flow”*^8^. The main functions of the cardiovascular system are to transport, control and maintain blood flow in the entire body. Abnormal hemodynamics greatly alters this tranquil picture, leading to initiation and progression of disease^9^. These abnormalities are often manifested by disturbed fluid dynamics^10^ (*local hemodynamics*), and in many cases by an increase in the heart workload (*global hemodynamics*). Hemodynamicsquantification can be greatly useful for accurate and early diagnosis but*we still lack proper diagnostic* methods for many cardiovascular diseases^11–13^ because the hemodynamics analysis methods that can be used as engines of new diagnostic tools are not well developed yet. Furthermore, as most interventions intend to recover the healthy condition, the ability to monitor and predict hemodynamics following particular interventions can have significant impacts on saving lives. Despite remarkable advances in medical imaging, imaging on its own is not predictive^11,14^. *Predictive methods are rare*. They are extensions of diagnostic methods, enabling prediction of effects of interventions, allowing timely and personalized interventions, and helping critical clinical decision makingabout life-threatening risks based on quantitative data.

The heart resides in a sophisticated vascular network whose loads impose boundary conditions on the heart function^6,7,14–16^. Effective diagnosis and prediction hinge on quantifications of the global hemodynamics (heart workload) and of the local hemodynamics (detailed information of the dynamics of the circulatory system, e.g., flow and pressure) of the cardiovascular system as all are very important for long-term health of the heart^6,14,16^. However, there is no method to invasively or noninvasively quantify the heart workload (global hemodynamics) and to provide contribution breakdown of each component of the cardiovascular diseases. Moreover, current diagnostic methods are limited and cannot quantify detailed information of the flow dynamics of the circulatory system (local hemodynamics). Although all of these can provide valuable information about the patient’s state of cardiac deterioration and heart recovery, currently, clinical decisions are chiefly made based on the anatomy alone. To augment anatomical information, cardiac catheterization is used as the clinical gold standard to evaluate pressure and flow through heart and circulatory system but it is invasive, expensive, high risk and therefore not practical for diagnosis in routine daily clinical practice or serial follow-up examinations^17^. Most importantly, cardiac catheterization only provides access to the blood pressure in very limited regions rather than details of the physiological pulsatile flow and pressures throughout the heart and the circulatory system. Phase-contrast magnetic resonance imaging can provide flow but it has poor temporal resolution, is costly, lengthy and not possible for many patients with implanted devices^18,19^. Doppler echocardiography (DE) is potentially the most versatile tool for hemodynamics as it is low-cost and risk-free and has a high temporal resolution. Despite all DE potentials and the progresses that have been made in its clinical use, to date, there have been no DE methods to comprehensively evaluate local hemodynamics, to evaluate global hemodynamics or to breakdown contributions of each components of the cardiovascular diseases. Computational mechanics has potentials to supplement DE to fill this gap and can offer a powerful means to augment clinical measurements to create non-invasive patient-specific diagnostic and predictive methods for monitoring, treatment planning and risk assessment.

In this study, an innovative non-invasive image-based patient-specific diagnostic, monitoring and predictive computational-mechanics frameworkwas was developed for C3VI. For simplicity, this C3VI computational mechanics framework is called C3VI-CMF in this paper. This computational tool enables (1) quantifying details of the physiological pulsatile flow and pressures through the heart and circulatory system (local hemodynamics); (2) quantifying heart function metrics, e.g., left ventricle workload (global hemodynamics). C3VI-CMF also provides the breakdown of effects of each disease constituents on the global function of the cardiovascular system. C3VI-CMF can also quantify other heart-function metrics such as the left-ventricular end-diastolic pressure and instantaneous left-ventricular pressure. *Currently, none of the above metrics can be obtained noninvasively in patients and when invasive procedures are undertaken, the collected metrics cannot be by any means as complete as the results that* C3VI-CMF *provides*. C3VI-CMF uses limited input parameters all of which can be measured using DE and sphygmomanometer. The tool only uses Doppler parameters that can be reliably measured. This tool has a lumped-parameter model at its core and includes several sub-models allowing analysis of any combination of complex valvular, vascular and ventricular diseases in both pre and post intervention conditions. In this paper, we report validation of C3VI-CMF against catheterization data in forty-nine patients with C3VI.

## Lumped parameter model

**Figure 1:**
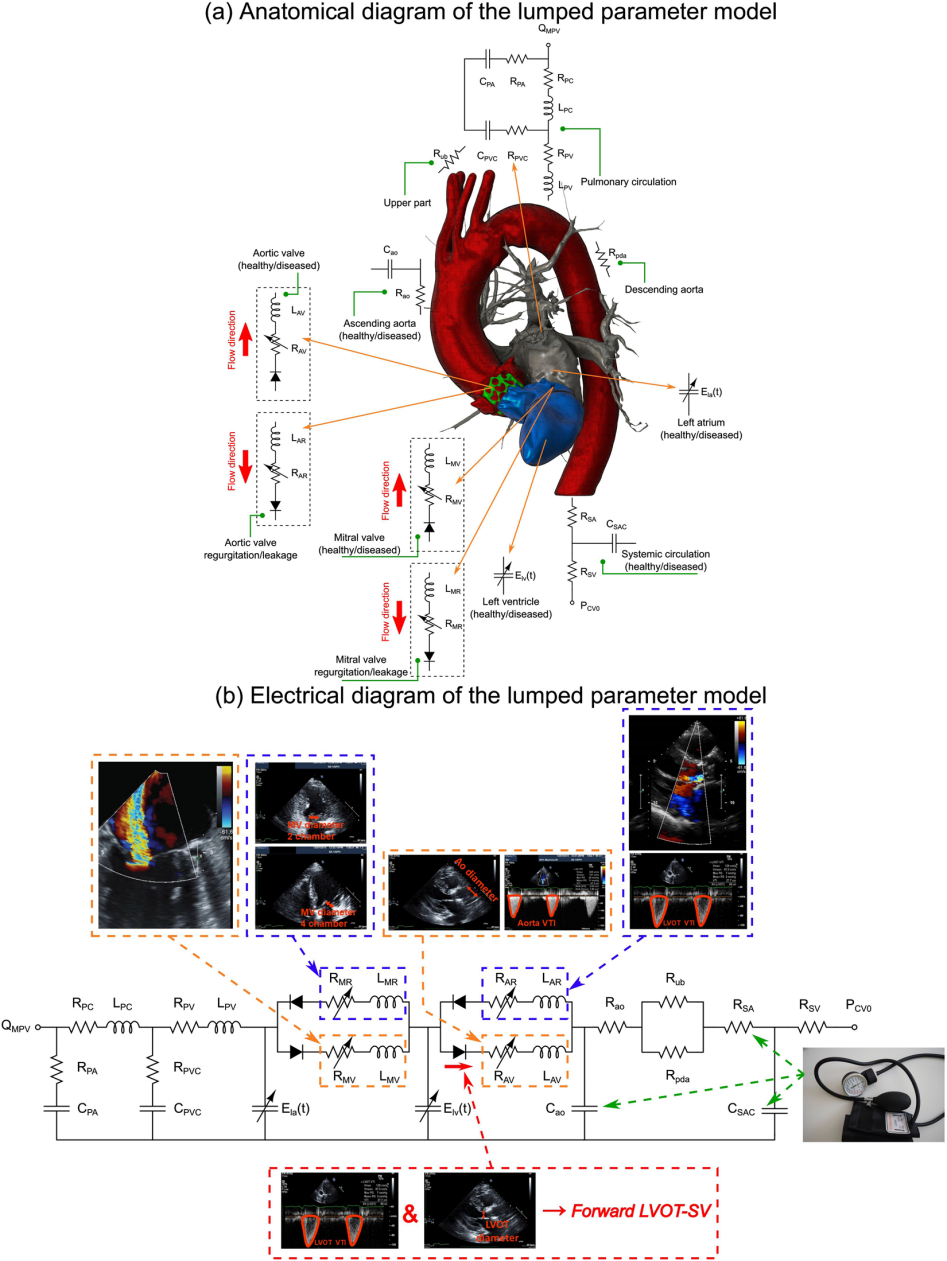
Schematic diagram of the lumped parameter modeling. (**a**) Anatomical representation; (**b**) Electrical representation. This model includes four sub-models. (1) left atrium, (2) left ventricle, (3) aortic valve, (4) mitral valve, (5) systemic circulation, and (6) pulmonary circulation. Abbreviations are similar as in Table 1. Input parameters were measured using Doppler echocardiography and sphygmomanometer. *Data Acquisition:* A computational mechanics framework based on non-invasive clinically measured hemodynamic metrics (brachial blood pressure and Doppler echocardiography measurements) was developed to estimate local and global hemodynamics.

**Figure 2:**
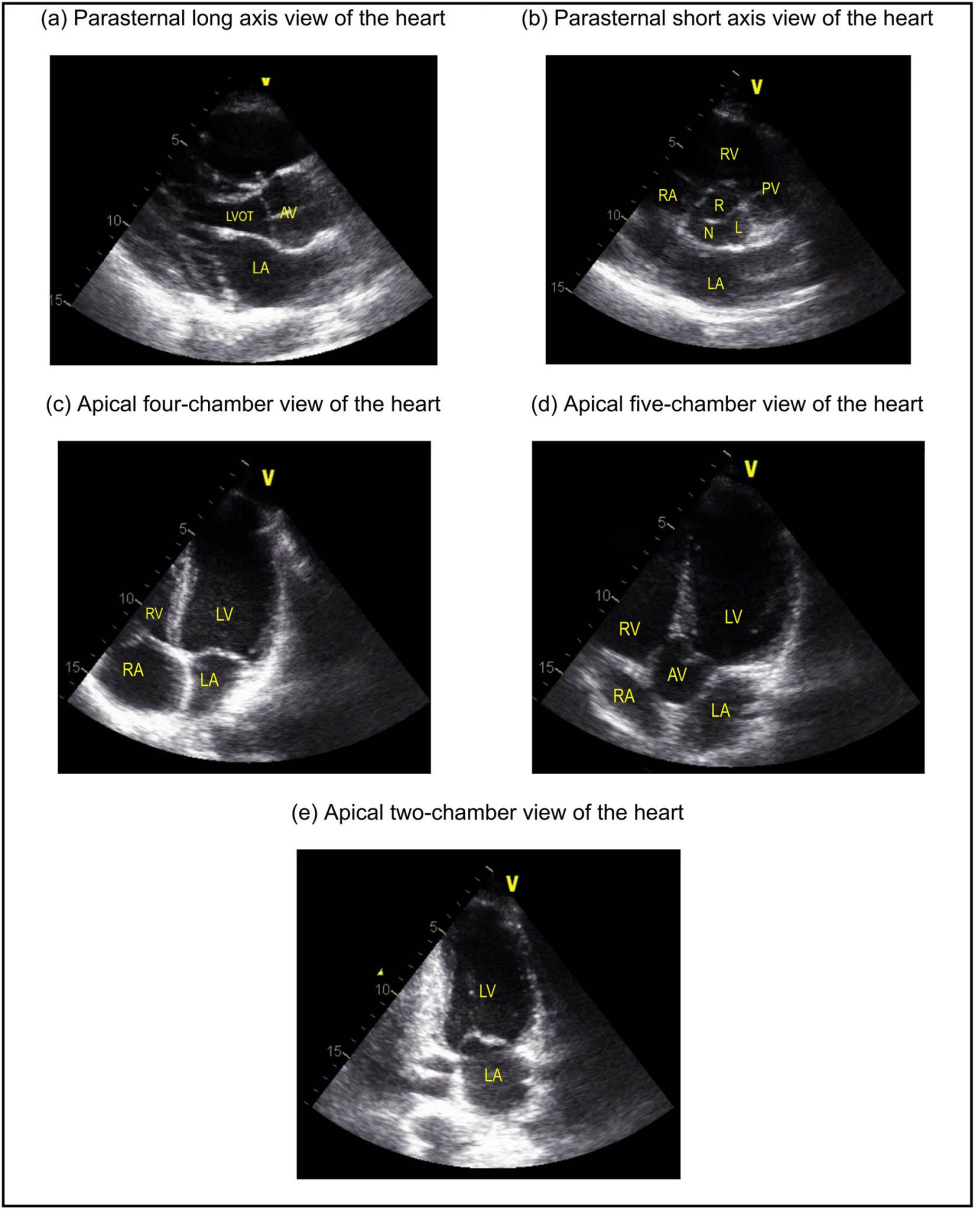
Views of heart used for Doppler echocardiography measurements. (**a**) Parasternal long axis view of the heart: blood enters the left ventricle through the left atrium, exiting through the left ventricular outflow tract leading to the aortic valve; (**b**) Parasternal short axis view of the heart: the aortic valve leaflets are shown opening and closing. Above the aortic valve is the right ventricle, through which blood exits the right ventricular outflow tract into the pulmonary artery; (**c**) Apical four-chamber view of the heart: right atrium opens into the right ventricle, and the left atrium opens into the left ventricle simultaneously; (**d**) Apical five-chamber view of the heart: mitral valve allows blood to enter the left ventricle, then exit through the aortic valve; (**e**) Apical two-chamber view of the heart: blood moves from the left atrium, through the mitral valve, into the left ventricle. Abbreviations: LVOT: left ventricular outflow tract; AV: aortic valve; LA: left atrium; RV: right ventricle; RA: right atrium; PV: pulmonary valve.

The developed algorithm (C3VI-CMF) consists of a parameter estimation algorithm (see below) and a lumped-parameter model that includes several sub-models allowing analysis of any combination of complex valvular, vascular and ventricular diseases in both pre and post intervention conditions: (1) left atrium, (2) left ventricle, (3) aortic valve, (4) mitral valve, (5) systemic circulation, and 6) pulmonary circulation (Fig. 1; Table 1). This paper reports an innovative method to integrate the parameter-estimation algorithm, the lumped-parameter model and non-invasive clinical Doppler echocardiography and sphygmomanometer measurements to make a patient-specific *in silico* model of the cardiovascular system. The algorithm uses the following input parameters that all can be reliably measured using Doppler echocardiography: forward left ventricular outflow tract stroke volume, heart rate, ejection time, ascending aorta area, left ventricular outflow tract area, aortic valve effective orifice area, mitral valve effective orifice area, and grading of aortic and mitral valves regurgitation severity. These parameters are measured in the parasternal long axis, parasternal short axis, apical two-chamber, apical four-chamber, and apical five-chamber views of the heart (Fig. 2). Other input parameters of the model are systolic and diastolic blood pressures measured using sphygmomanometers. Note that the proposed method does not need any catheter data as input parameters of the model. This innovative lumped-parameter model calculations were validated against cardiac catheterization data (the instantaneous pressures in the aorta and LV) in forty-nine patients with C3VI (see Results section for validation, Table 1 for patient-specific input parameters and Table 2 for patient’s characteristics). Two sub-models (aortic stenosis and aortic regurgitation) have already been used^7,20,21^ and validated against *in vivo* cardiac catheterization (N = 34)^15^ and *in vivo* MRI data (N = 57)^22^.

**Table 1:** Cardiovascular parameters. Summarized parameters used in the lumped parameter modeling to simulate all patient-specific cases.

Description	Abbreviation	Value		
**Valve parameters**				
Effective orifice area	EOA	Measured using DE		
Energy loss coefficient	E_L_Co	$\frac{(EOA)A}{A-EOA}$; EOA and A are measured using DE		
Variable resistance	R_AV_ & R_AR_	$\frac{\rho }{2{{E}_{L}Co|}_{AV}^{2}}Q(t)$	&	$\frac{\rho }{2{{E}_{L}Co|}_{AR}^{2}}Q(t)$
	R_MV_ & R_MR_	$\frac{\rho }{2{EOA|}_{MV}^{2}}{Q}_{MV}(t)$	&	$\frac{\rho }{2{EOA|}_{MR}^{2}}Q(t)$
Inductance	L_AV_ & L_AR_	$\frac{2\pi \rho }{\sqrt{{{E}_{L}Co|}_{AV}}}$	&	$\frac{2\pi \rho }{\sqrt{{{E}_{L}Co|}_{AR}}}$
	L_MV_ & L_MR_	$\frac{{M}_{MV}}{EO{A}_{MV}}$	&	$\frac{{M}_{MV}}{EO{A}_{MR}}$
Inertance (mitral valve)	M_MV_	Constant value: 0.53 gcm^−2^		
**Systematic circulation parameters**				
Aortic resistance	R_ao_	Constant value: 0.05 mmHg.s.mL^−1^		
Aortic compliance	C_ao_	Initial value: 0.5 mL/mmHg Optimized based on brachial pressures *(Systolic and diastolic brachial pressures are optimization constraints)*		
Systemic vein resistance	R_SV_	0.05 mmHg.s.mL^−1^		
Systemic arteries and veins compliance	C_SAC_	Initial value: 2 mL/mmHg Optimized based on brachial pressures *(Systolic and diastolic brachial pressures are optimization constraints)*		
systemic arteries resistance (including arteries, arterioles and capillaries)	R_SA_	Initial value: 0.8 mmHg.s.mL^−1^ Optimized based on brachial pressures *(Systolic and diastolic brachial pressures are optimization constraints)*		
Upper body resistance	R_ub_	Adjusted to have 15% of total flow rate in healthy case^15^		
Proximal descending aorta resistance	R_pda_	Constant value: 0.05 mmHg.s.mL^−1^		
Elastance Function^*^				
Maximum elastance	E_max_	2.1 (LV) 0.17 (LA)		
Minimum elastance	E_min_	0.06 (LV, LA)		
Elastance ascending gradient	m_1_	1.32 (LV, LA)		
Elastance descending gradient	m_2_	27.4 (LV) 13.1 (LA)		
Elastance ascending time translation	*τ*_1_	0.269 T (LV) 0.110 T (LA)		
Elastance descending time translation	*τ*_2_	0.452 T (LV) 0.18 T (LA)		
Elastance normalization	N	$\frac{{E}_{MAX}-{E}_{MIN}}{2}$		
**Pulmonary circulation parameters**				
Pulmonary vein inertance	L_PV_	Constant value:0.0005 mmHg·s^2^·mL^−1^		
Pulmonary vein resistance	R_PV_	Constant value: 0.002 mmHg·s·mL^−1^		
Pulmonary vein and capillary resistance	R_PVC_	Constant value: 0.001 mmHg·s·mL^−1^		
Pulmonary vein and capillary compliance	C_PVC_	Constant value: 40 mL/mmHg		
Pulmonary capillary inertance	L_PC_	Constant value: 0.0003 mmHg·s^2^·mL^−1^		
Pulmonary capillary resistance	R_PC_	Constant value: 0.21 mmHg·s·mL^−1^		
Pulmonary arterial resistance	R_PA_	Constant value: 0.01 mmHg·s·mL^−1^		
Pulmonary arterial compliance	C_PA_	Constant value: 4 mL/mmHg		
Mean flow rate of pulmonary valve	Q_MPV_	*Forward LVOT-SV*is the only input flow condition (measured using DE). *Q*_*MPV*_* is a flow parameter that was optimized so that the lumped- parameter model could reproduce the desirable DE-measured Forward LVOT-SV*.		
**Input flow condition**				
Forward left ventricular outflow tract stroke volume	Forward LVOT-SV	Measured using DE		
**Output condition**				
Central venous pressure	P_CV0_	Constant value: 4 mmHg		
**Other**				
Blood density	$\rho$	Constant value: 1050 kg/m^3^		
Heart rate	HR	Measured using DE		
Duration of cardiac cycle	T	Measured using DE		
Systolic end ejection time	T_EJ_	Measured using DE		
End diastolic volume	EDV	Measured using DE		
End systolic volume	ESV	Measured using DE

**Table 2:** Baseline patient characteristics. Changes in hemodynamic metrics from baseline to 90-day post-TAVR.

	Pre intervention; Mean ± SD (n = 49)	90-day post intervention; Mean ± SD (n = 49)
**Ventricular indices – DE findings**		
Ejection fraction, %	53.5 ± 12.7	61 ± 14.6
Heart rate, bpm	70.7 ± 9.5	68 ± 11.8
Stroke volume, mL	48.3 ± 11.7	44.5 ± 15.5
**Valvular indices – DE findings**		
Aortic valve effective orifice area (cm^2^)	0.58 ± 0.16	1.75 ± 0.4
Mean aortic valve gradient, mmHg	51.52 ± 13.6	11.1 ± 6.1
Maximum aortic valve gradient, mmHg	84.5 ± 21.32	20.4 ± 10.28
Aortic valve disease type	Tricuspid: 45; Bicuspid: 4	N/A
Transcatheter valve prosthetic size, mm	N/A	26.87 ± 1.6
Transcatheter valve prosthetic type	N/A	CoreValve, SAPIEN & SAPIEN XT
Aortic valve Regurgitation ≥ grade 2	48%	5%
Mitral valve Regurgitation ≥ grade 2	19%	20%
**Vascular indices – Sphygmomanometer**		
Brachial systolic blood pressure, mmHg	139 ± 22.5	135 ± 16.8
Brachial diastolic blood pressure, mmHg	79 ± 11.7	68 ± 10.3
**Patient description**		
Mean age, years; Gender	64.5 ± 5.5; (Female: 36%)	N/A
Mean weight, kg; Mean height, cm	73.4 ± 12.8; 165.7 ± 9.6	N/A
Body surface area, m^2^	1.73 ± 0.14	N/A
Body mass index, kg/m^2^	31.9 ± 21.5	N/A

### Heart-arterial model

#### Left ventricle

Coupling between LV pressure and volume was performed through a time varying elastance E(t), a measure of cardiac muscle stiffness.

1 $$E(t)=\frac{d{P}_{LV}(t)}{V(t)-{V}_{0}}$$

where ${P}_{LV}(t)$, $V(t)$ and ${V}_{0}$ are left ventricle time-varying pressure, time-varying volume and unloaded volume, respectively^15^. The amplitude of E(t) can be normalized with respect to maximal elastance E_max_, *i.e*., the slope of the end-systolic pressure-volume relation, giving E_N_(t_N_)=E(t)/E_max_. Time then can be normalized with respect to the time to reach peak elastance, T_Emax_ (t_N_ = t/T_Emax_).

2 $${E}_{\max }{E}_{N}(t/{T}_{E\max })=\frac{d{P}_{LV}(t)}{V(t)-{V}_{0}}$$

To model the normalized elastance function of the LV, we tried three functions: (1) a summation of Gaussian functions^23,24^, (2) a Boltzmann Distribution^25^, and (3) a double Hill function^26,27^. We simulated the lumped-parameter model using these elastance functions for several different patient input parameters and found that the double Hill function model gave the most accurate (physiologically realistic) results for the pressure, flow, and volume waveforms. The use of the double Hill function was motivated by myocyte recruitment during preload, which is fundamentally a cooperative process^28^ and consequently, is modeled by a sigmoidal Hill function^29^. Both the Gaussian function and Boltzmann distribution not only gave sub-par results compared to the Hill model, but also did not model the myocyte recruitment mechanism: The Gaussian function is symmetric about a mean^23^, which is not correct for our model because contraction and relaxation are not symmetric processes^30–39^. The Boltzmann distribution is a probability distribution of physical states^25^, and hence does not capture the dynamic cooperativity of myocytes recruitment. Consequently, to model the LV normalized time-varying elastance curves (E_N_), we used a double Hill function as the following^26,27^:

3 $${E}_{N}(t)=N\left(\frac{{\left(\frac{t}{{\tau }_{1}}\right)}^{m1}}{1+{\left(\frac{t}{{\tau }_{1}}\right)}^{m1}}\right)\left(\frac{1}{1+{\left(\frac{t}{{\tau }_{2}}\right)}^{m2}}\right)+{E}_{\min }$$

where $N$, ${\tau }_{1}$, ${\tau }_{2}$, ${m}_{1}$, ${m}_{2}$, and ${E}_{\min }$ are elastane normalization, ascending time translation, descending time translation, ascending gradient, descending gradient, and minimum elastance, respectively (see Table 1). A double Hill function was deemed necessary to model the contraction and relaxation in the heart chambers: in Eq. 3, the first term in brackets corresponds to the contraction of the chamber and the second term in brackets corresponds to the relaxation of the chamber. ${\tau }_{1}$, ${\tau }_{2}$, ${m}_{1}$, ${m}_{2}$ govern the time translation and gradient of the elastance function, respectively. Parameter values used for the elastance function were adapted from^30–39^ to obtain physiologically realistic waveforms for pressure, volume, and flow (See Table 1).

#### Left atrium

Coupling between LA pressure and volume was performed through a time varying elastance E(t), a measure of cardiac muscle stiffness, using the same procedure as outlined above for the LV. The elastance function used for the LA is as defined in Eqs. 2 and 3^26,27^; parameter values used can be found in Table 1. Additionally, to take into account the relative onset of contraction for the LA and LV, a phase lag was used in the LA elastance function^26^. Specifically, LV contraction was initiated at T = 0, and LA contraction was initiated at 0.85 T^26^, resulting in a time delay of 0.15 T.

### Modeling heart valves

#### Modeling aortic valve

##### Aortic valve.

Aortic valve was modeled using the net pressure gradient formulation $(P{G}_{net})$ across the aortic valve during LV ejection. This formulation expresses the instantaneous net pressure gradient across the aortic valve (after pressure recovery) as a function of the instantaneous flow rate and the energy loss coefficient and links the LV pressure to the ascending aorta pressure:

4 $${P{G}_{net}|}_{AV}=\frac{2\pi \rho }{\sqrt{{{E}_{L}Co|}_{AV}}}\frac{\partial Q(t)}{\partial t}+\frac{\rho }{2{{E}_{L}Co|}_{AV}^{2}}{Q}^{2}(t)$$

and

5 $${{E}_{L}Co|}_{AV}=\frac{({EOA|}_{AV}){A}_{AO}}{A-{EOA|}_{AV}}$$

where ${{E}_{L}Co|}_{AV}$, ${EOA|}_{AV}$, ${A}_{AO}$, $\rho$ and $Q$ are the valvular energy loss coefficient, the effective orifice area, ascending aorta cross sectional area, fluid density and transvalvular flow rate, respectively. ${{E}_{L}Co|}_{AV}$, representing the ‘recovered EOA’, denotes valve effective orifice area adjusted for the area of the aorta at the level of sinotubular junction.

##### Aortic regurgitation.

Aortic regurgitation (AR) was modeled using the same analytical formulation as aortic stenosis as the following. AR pressure gradient is the difference between aortic pressure and LV pressure during diastole.

6A $${P{G}_{net}|}_{AR}=\frac{2\pi \rho }{\sqrt{{{E}_{L}Co|}_{AR}}}\frac{\partial Q(t)}{\partial t}+\frac{\rho }{2{{E}_{L}Co|}_{AR}^{2}}{Q}^{2}(t)$$

and

6B $${{E}_{L}Co|}_{AR}=\frac{EO{A}_{AR}{A}_{LVOT}}{{A}_{LVOT}-EO{A}_{AR}}$$

where ${{E}_{L}Co|}_{AR}$, $EO{A}_{AR}$ and ${A}_{LVOT}$ are regurgitation energy loss coefficient, regurgitant effective orifice area and LVOT area, respectively.

#### Modeling mitral valve

##### Mitral valve.

Mitral valve (MV) was modeled using the analytical formulation for the net pressure gradient (${P{G}_{net}|}_{MV}$) across the MV during LA ejection. This formulation expresses the instantaneous net pressure gradient across the LA and vena contracta as an unsteady incompressible inviscid flow, where viscous effect is ignored, with a constant blood density. ${P{G}_{net}|}_{MV}$ expresses as a function of $\rho$, ${Q}_{MV}$, $EO{A}_{MV}$ and ${M}_{MV}$ where these quantities represent the density of fluid, transvalvular flow rate, effective orifice area and inertance, respectively. In this formulation, the pressure recovery phenomenon was ignored because the effect is negligible due to the large volume of the LV^40^.

7 $${P{G}_{net}|}_{MV}=\frac{{M}_{MV}}{EO{A}_{MV}}\frac{\partial {Q}_{MV}(t)}{\partial t}+\frac{\rho }{2{EOA|}_{MV}^{2}}{{Q}_{MV}}^{2}(t)$$

##### Mitral regurgitation

Mitral regurgitation (MR) was modeled using Eq. 8. MR pressure gradient is the difference between mitral pressure and LA pressure during systole.

8 $${P{G}_{net}|}_{MR}=\frac{{M}_{MV}}{EO{A}_{MR}}\frac{\partial Q(t)}{\partial t}+\frac{\rho }{2{EOA|}_{MR}^{2}}{Q}^{2}(t)$$

where ${EOA|}_{MR}$ is MR effective orifice area.

#### Pulmonary flow

The pulmonary valve flow waveform was simulated by a rectified sine curve with duration ${t}_{ee}$ and amplitude Q_MPV_ as the following.

9 $${Q}_{PV}(t)={Q}_{MPV}\,\sin \left(\frac{\pi t}{{t}_{ee}}\right),t\le {t}_{ee};{Q}_{PV}(t)=0,{t}_{ee} < t\le T$$

where Q_MPV_, t_ee_ and T are mean flow rate of the pulmonary valve, end-ejection time and cardiac cycle time period, respectively. In this study, Forward LVOT-SV is the only input flow condition which is reliable to measure using DE. Q_MPV_, the mean flow rate of the pulmonary valve, was optimized so that the lump-parameter model could reproduce the desirable DE-measured Forward LVOT-SV.

### Determining arterial compliance and peripheral resistance

The total systemic resistance was computed as the quotient of the average brachial pressure and the cardiac output (assuming a negligible peripheral venous pressure (mean ~ 5 mmHg) compared to aortic pressure (mean ~ 100 mmHg). This total systemic resistance represents the electrical equivalent resistance for all resistances in the current model. Because what the left ventricle faces is the total systemic resistance and not the individual resistances, for the sake of simplicity we considered the aortic resistance, ${R}_{ao}$, and systemic vein resistance, ${R}_{SV}$, as constants and adjusted the systemic artery resistance,${R}_{SA}$, according to the obtained total systemic resistance. Systemic artery resistance was evaluated using an optimization scheme outlined in the patient-specific parameter estimation section.

Physiologically, arterial hypertension is determined by two factors: the degree of reduction in the caliber of small arteries or arterioles with an ensuing increase in systemic vascular resistance and mean blood pressure, and the extent of reduction in the arterial compliance with a resulting increase in pulse pressure (systolic minus diastolic blood pressure). For each degree of hypertension, we fit the predicted pulse pressure to the actual pulse pressure (known by arm cuff sphygmomanometer) obtained from clinical study by adjusting compliances (aorta (C_ao_) and systemic (C_SAC_)). Therefore, for each degree of arterial hypertension, the compliance was evaluated using an optimization scheme outlined in the patient-specific parameter estimation section.

### Patient-specific parameter estimation

**Figure 3:**
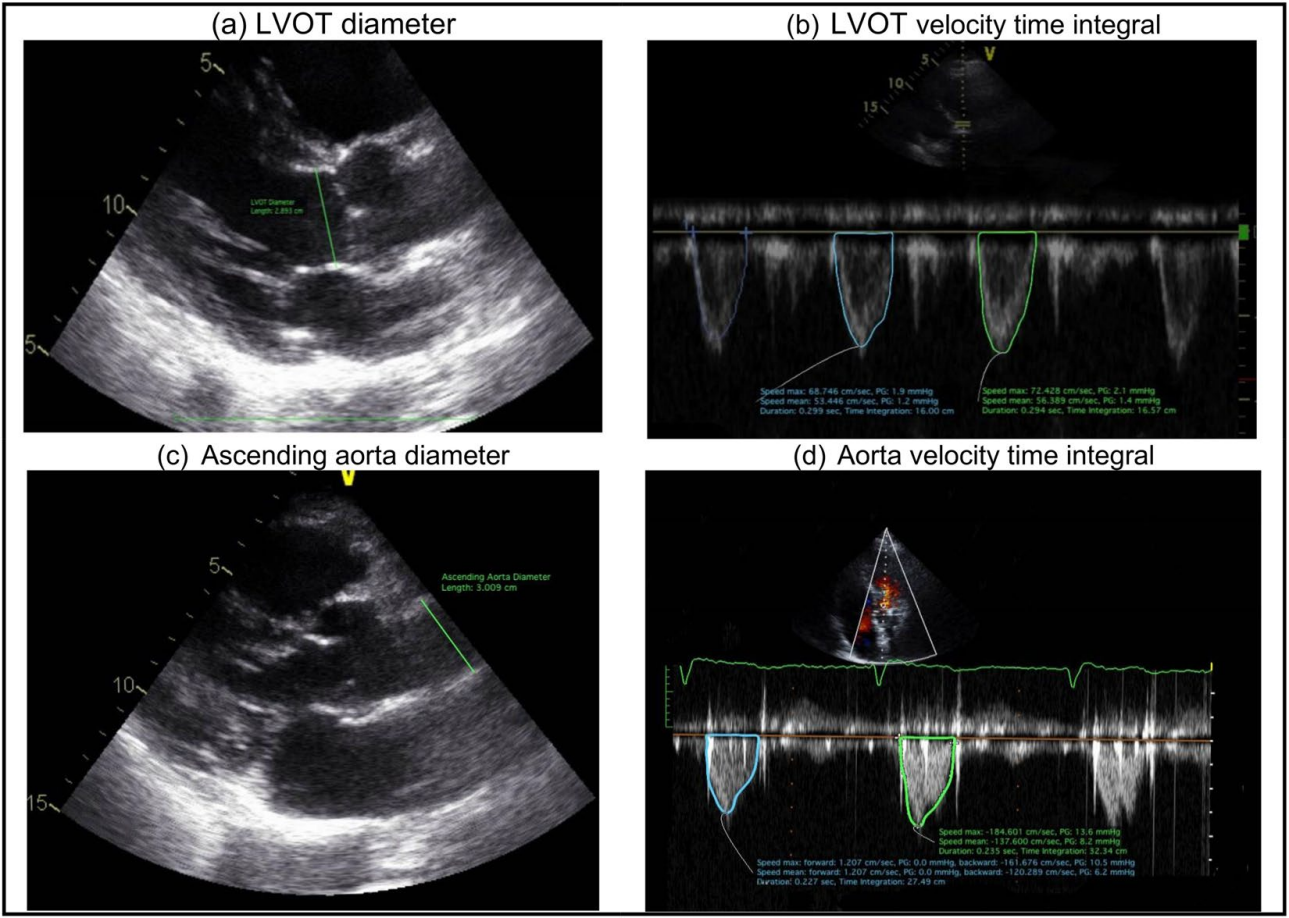
Doppler echocardiography measurements for left ventricular outflow tract and the aorta. (**a**) Left ventricular outflow tract diameter, measured in the parasternal long axis view; (**b**) left ventricular outflow tract velocity time integral, taken as the average of the areas; (**c**) Ascending aorta diameter, measured in the parasternal long axis view; (**d**) Aorta velocity time integral, taken as the average of the areas.

The lumped-parameter model took the following patient-specific parameters as its inputs: forward left ventricular outflow tract stroke volume (*Forward LVOT-SV*), cardiac cycle time (T), ejection time (T_EJ_), EOA_AV_, EOA_MV_, A_AO_, A_LVOT_, EOA_AR_, EOA_MR_ and brachial systolic and diastolic pressures measured by a sphygmomanometer. The following procedure was used to set up the patient-specific lumped-parameter model in the following sequence:

***1) Flow inputs***: The lumped-parameter model used only one reliably measured flow parameter as an input: forward left-ventricular outflow tract stroke volume (*Forward LVOT-SV*) (Eq. 10). *Forward LVOT-SV* is defined as the volume of blood that passes through the LVOT cross sectional area every time the heart beats.

10 $$Forward\,{LVOT}-{SV}={A}_{LVOT}\times VT{I}_{LVOT}=\frac{\pi \times {({D}_{LVOT})}^{2}}{4}\times VT{I}_{LVOT}$$

where ${D}_{LVOT}$, ${A}_{LVOT}$, and $VT{I}_{LVOT}$ are LVOT diameter, LVOT area, and LVOT velocity-time integral, respectively, all reliably measured using Doppler echocardiography (Fig. 3).

***2) Time inputs***: Cardiac cycle time (T) and ejection time (T_EJ_) were measured using Doppler echocardiography.

***3) Aortic valve inputs***: ${A}_{AO}$ and ${EOA|}_{AV}$ were calculated using Eqs. 11 and 12, respectively.

11 $${A}_{AO}=\frac{\pi \times {({D}_{AO})}^{2}}{4}$$

12 $${EOA|}_{AV}=\frac{{ForwardLVOT}-{SV}}{VT{I}_{AO}}$$

where ${D}_{AO}$ and $VT{I}_{AO}$ are the diameter of the ascending aorta and velocity time integral in the ascending aorta, respectively (Fig. 3). $VT{I}_{AO}$ is the amount of the blood flow going through the aorta which was obtained by tracing the aorta pulse wave flow Doppler envelope (Fig. 3). To model the blood flow in the forward direction, ${A}_{AO}$ and ${EOA|}_{AV}$ were then substituted into Eq. (4) and the constant inductance ($\frac{2\pi \rho }{\sqrt{{{E}_{L}Co|}_{AV}}}$) and variable resistance ($\frac{\rho }{2{{E}_{L}Co|}_{AV}^{2}}Q(t)$) parameters were calculated.

**Figure 4:**
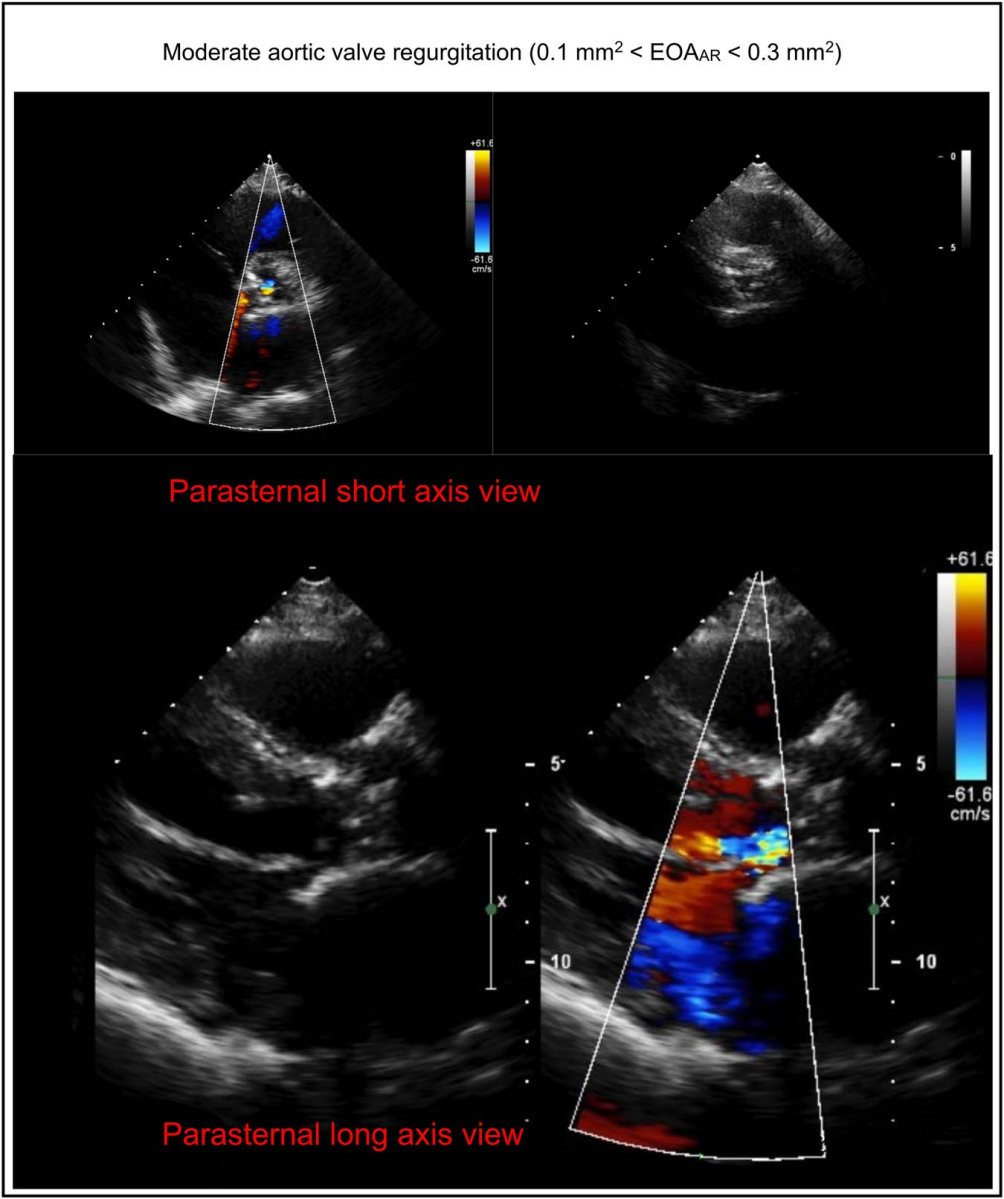
Doppler echocardiography investigation for aortic valve regurgitation. To evaluate aortic valve regurgitation severity, aortic valve color Doppler images are used in both long axis, and short axis views. This image is an example of moderate to severe aortic valve regurgitation in a patient with AS who received TAVR (0.2 mm^2^ < EOA_AR_ < 0.3 mm^2^).

***4) Aortic regurgitation inputs***: To model blood flow in the reverse direction (aortic valve insufficiency), *EOA*_*AR*_ and ${A}_{LVOT}$ were substituted into Equation (6) to calculate the variable resistance ($\frac{\rho }{2{{E}_{L}Co|}_{AR}^{2}}Q(t)$) and constant inductance ($\frac{2\pi \rho }{\sqrt{{{E}_{L}Co|}_{AR}}}$) parameters. For patients with no insufficiency, the reverse branch is not included. ${A}_{LVOT}$ was quantified using Doppler echocardiography measurements (Fig. 3). The *EOA*_*AR*_ can be calculated by dividing the regurgitant volume by the time-velocity integral of regurgitant flow using continuous wave Doppler. However, such a calculation does not always yield a correct *EOA*_*AR*_ and therefore is not deemed to be reliable. Therefore, to quantify Doppler aortic regurgitant effective orifice area (*EOA*_*AR*_), aortic valve regurgitation was investigated using color Doppler images in both the long axis and short axis views by experienced cardiologists and graded qualitatively as either mild regurgitation (equivalent to EOA_AR_ < 0.1 mm^2^), mild to moderate regurgitation (equivalent to 0.1 mm^2^ < EOA_AR_ < 0.2 mm^2^), moderate to severe regurgitation (equivalent to 0.2 mm^2^ < EOA_AR_ < 0.3 mm^2^), or severe regurgitation (equivalent to EOA_AR_ > 0.3 mm^2^) (see Fig. 4 for an example of moderate to severe aortic valve regurgitation in a patient with AS who received TAVR)^41,42^.

**Figure 5:**
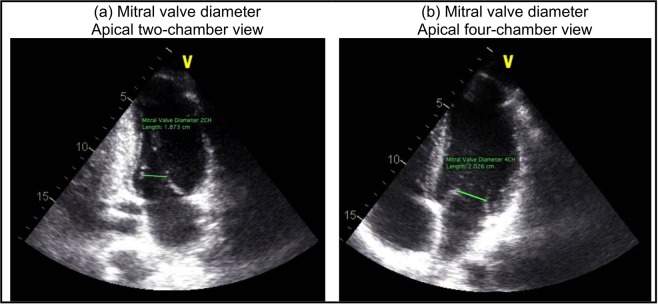
Mitral valve dimensions. (**a**) Mitral valve diameter (d_1_), measured in apical two-chamber view; (**b**) Mitral valve diameter (d_2_), measured in apical four-chamber view. Mitral valve is an ellipse and its area is quantified using A_MV_ =$\frac{\pi \ast {d}_{1}\ast {d}_{2}}{4}$.

***5) Mitral valve inputs***: To model the blood flow in the forward direction, mitral valve area was substituted into Eq. (8) and the constant inductance ($\frac{{M}_{MV}}{EO{A}_{MV}}$) and variable resistance ($\frac{\rho }{{2EOA|}_{MV}^{2}}{Q}_{MV}(t)$) parameters were calculated. Mitral valve is approximately an ellipse and its area was quantified using A_MV_ =$\frac{\pi \ast {d}_{1}\ast {d}_{2}}{4}$ where d_1_ and d_2_ are mitral-valve diameters measured in the apical two-chamber and apical four-chamber views, respectively (Fig. 5).

**Figure 6:**
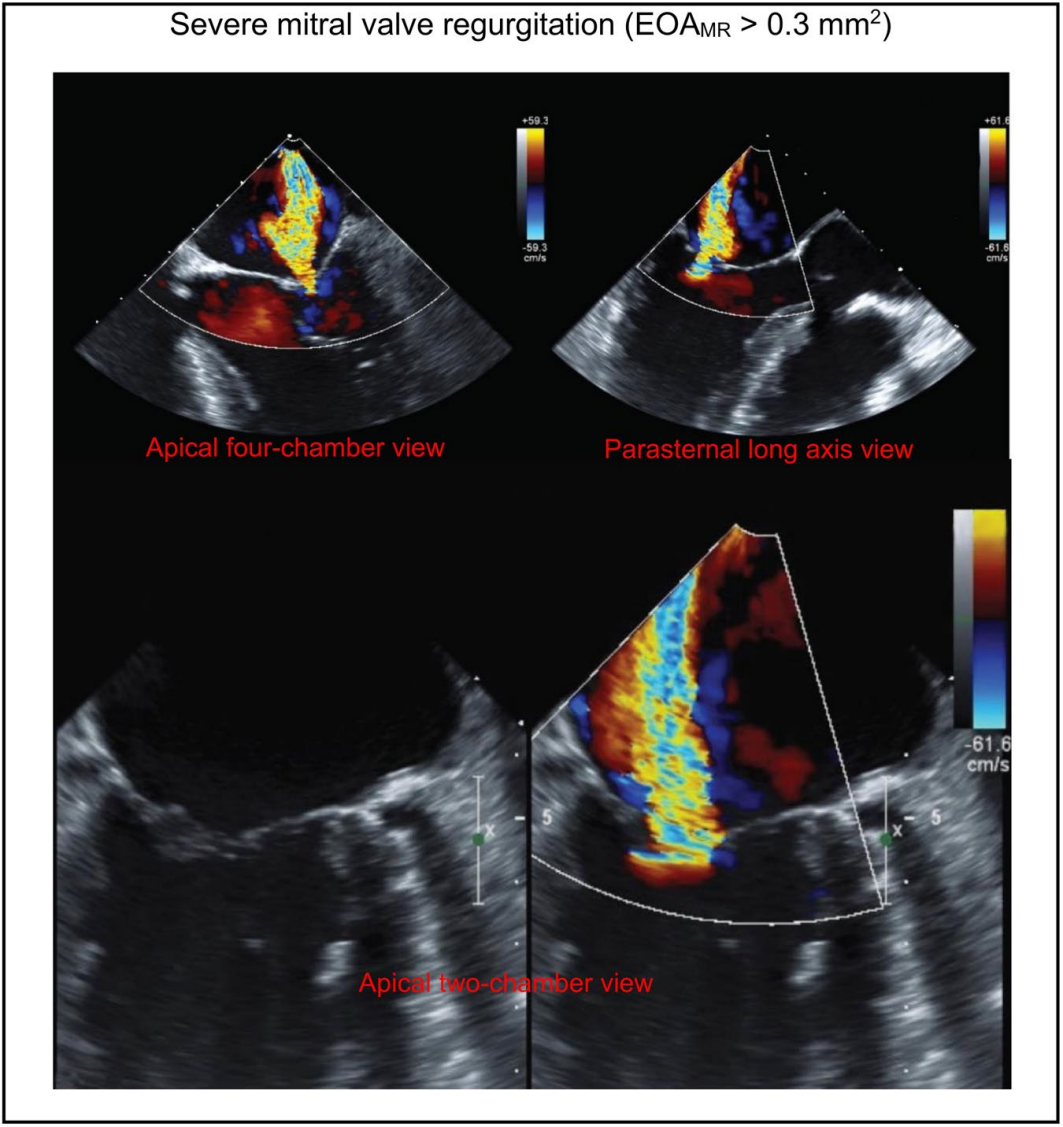
Doppler echocardiography investigation for mitral valve regurgitation. To evaluate mitral valve regurgitation severity, mitral valve color Doppler images are used in apical four-chamber view (top left), parasternal long axis view (top right), and apical two-chamber view (bottom). The three images used are of the same patient, and each demonstrates sever mitral valve regurgitation. This figure is an example of severe mitral valve regurgitation in a patient with AS who received TAVR  EOA_MR_ > 0.3 mm^2^).

***6) Mitral regurgitation inputs***: To model blood flow in the reverse direction (mitral-valve insufficiency), EOA_MR_ is substituted into Eq. (9) to calculate the variable resistance ($\frac{\rho }{{2EOA|}_{MV}^{2}}Q(t)$) and constant inductance ($\frac{{M}_{MV}}{EO{A}_{MR}}$) parameters. For patients with no insufficiency, the reverse branch was not included. As described for the aortic-valve regurgitation, calculation of the regurgitant effective orifice area by dividing the regurgitant volume by the time-velocity integral of regurgitant flow using continuous wave Doppler is not reliable. Therefore, to quantify mitral regurgitant effective orifice area (EOA_MR_), mitral valve regurgitation was investigated using color Doppler images in the apical four-chamber, parasternal long axis, and apical two-chamber views by experienced cardiologists and graded qualitatively as either mild regurgitation (equivalent to EOA_MR_ < 0.1 mm^2^), mild to moderate regurgitation (equivalent to 0.1 mm^2^ < EOA_MR_ < 0.2 mm^2^), moderate to severe regurgitation (equivalent to 0.2 mm^2^ < EOA_MR_ < 0.3 mm^2^), or severe regurgitation (equivalent to EOA_MR_ > 0.3 mm^2^) (see Fig. 6 for an example of severe mitral-valve regurgitation in a patient who received TAVR).

**Figure 7:**
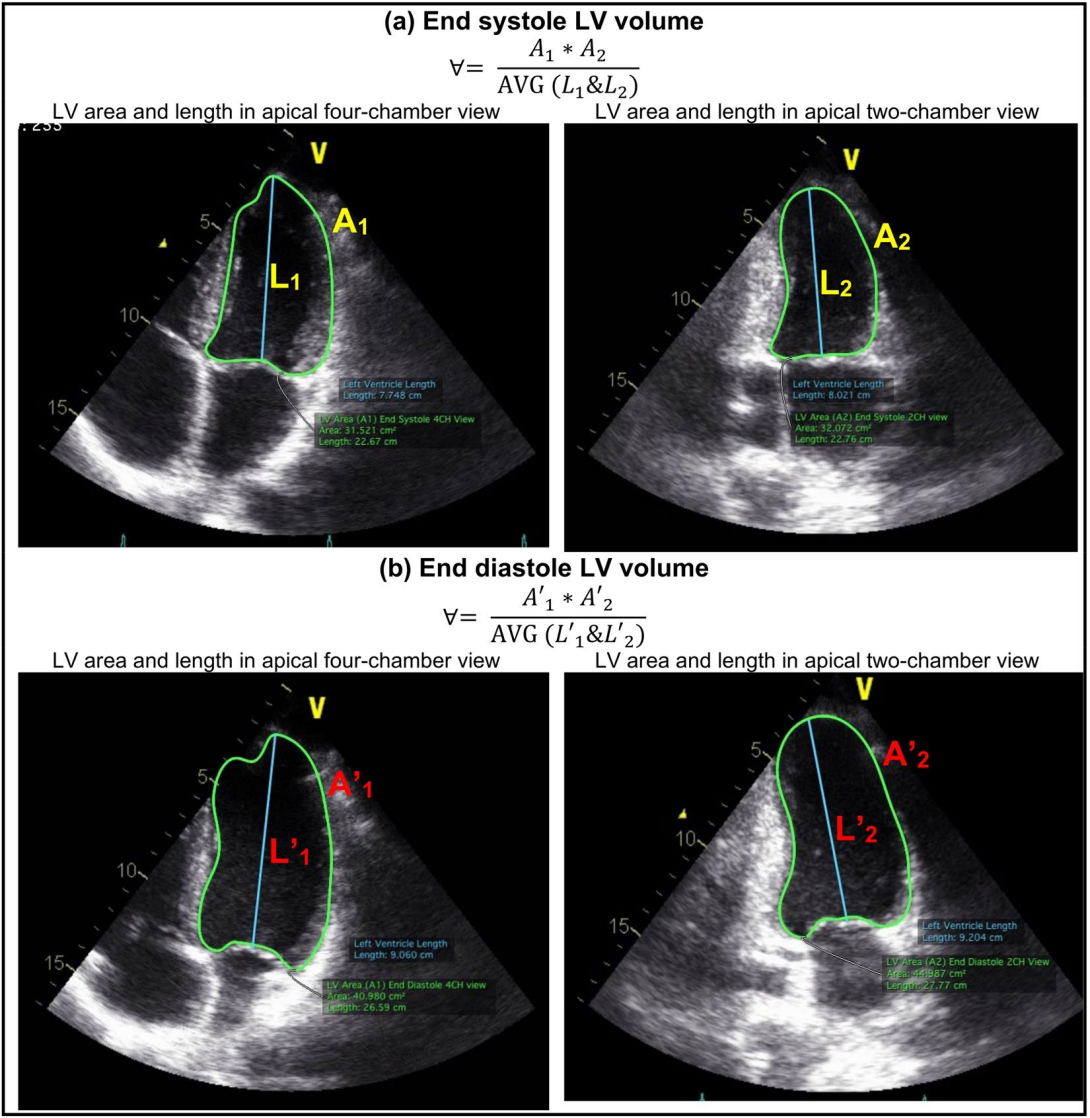
LV volumes. (**a**) End of systole LV volume; (**b**) End of diastole LV volume.

***7) End systolic volume and end diastolic volume***: End systolic volume (ESV) or end diastolic volume (EDV) measured using Doppler echocardiography was fed to the lumped-parameter model to adjust starting and ending volumes in the P-V loop diagram. For this purpose, the Biplane Ellipsoid model was used to calculate the instantaneous LV volume at the end of diastole and the end of systole using the following Equation.

13 $$\forall =\frac{{A}_{1}\,\ast \,{A}_{2}}{{\rm{AVG}}({L}_{1}\& {L}_{2})}$$

where A_1_, A_2_, L_1_, L_2_ and AVG (L_1_&L_2_) are LV area measured in the apical four-chamber view, LV area measured in the apical two-chamber view, LV length measured in the apical four-chamber view, LV length measured in the apical two-chamber view, and average of these two LV lengths, respectively (Refer to Fig. 7 for an example).

Ejection Fraction was then calculated as follow:

14 $${\rm{EF}}=\frac{{\rm{EDV}}-{\rm{ESV}}}{{\rm{EDV}}}$$

***8) Left-ventricle inputs:*** The cardiac cycle time (T) was substituted into ${\tau }_{1}$, ${\tau }_{2}$, ${m}_{1}$ and ${m}_{2}$ in Table 1 and then those values were substituted into Equation 3 to determine the elastance function.

***9) Left-atrium inputs:*** The cardiac cycle time (T) was substituted into ${\tau }_{1}$, ${\tau }_{2}$, ${m}_{1}$ and ${m}_{2}$ in Table 1 and then those values were substituted into Equation 3 to determine the elastance function.

***10) Parameter estimation for systemic circulation****:* Parameters R_SA_, C_SVC_, and C_ao_were optimized so that the aorta pressure calculated using the model matched the patient’s systolic and diastolic brachial pressures measured using a sphygmomanometer (see computational algorithm section for details). The initial values of these parameters are given in Table 1.

***11) Simulation execution***: Please see the computational algorithm section.

### Computational algorithm

The lumped-parameter model was analyzed numerically by creating and solving a system of ordinary differential equations in Matlab Simscape (MathWorks, Inc.), enhanced by adding additional functions written in Matlab and Simscape. Matlab’s ode23t trapezoidal rule variable-step solver was used to solve the system of differential equations with an initial time step of 0.1 milliseconds. The convergence residual criterion was set to 10^−6^ and initial voltages and currents of capacitors and inductors were set to zero. The model was run for several cycles to reach steady state before starting the response optimization process, described below.

A double Hill function representation of a normalized elastance curve for human adults^26,27^ was used to generate a signal to model LV elastance. It was shown that this elastance formulation can correctly represent the LV function independent from its healthy and/or pathological conditions. Simulations started at the onset of isovolumic contraction. The instantaneous LV volume, V(t), was calculated using the LV pressure, P_LV_, and the time varying elastance (Eq. 1). The LV flow rate was subsequently calculated as the time derivative of the instantaneous LV volume. The same approach was used to obtain the left-atrium volume, pressure and flow rate. P_LV_ was first calculated using the initial values of the model input parameters from Table 1. The *Forward LVOT-SV* calculated using the lumped-parameter model was then fitted to the one measured (Equation10) by optimizing Q_MPV_ (as detailed below). Finally, for each patient, R_SA_, C_SVC_, and C_ao_ were optimized to fit the aorta pressure from the model to the patient systolic and diastolic pressures measured using a sphygmomanometer.

### Patient-specific response optimization

In order to correctly simulate the conditions of the body of each patient, some of the parameters of the model were optimized so that the lumped-parameter model reproduced the physiological measurements performed in the patient. We conducted an extensive parameter sensitivity analysis that revealed negligible effects of changes in the pulmonary parameters (e.g., C_PVC_) on the model output variables. We, therefore, did not include these pulmonary parameters in the parameter-identification process and used the values given in Table 1.

**Figure 8:**
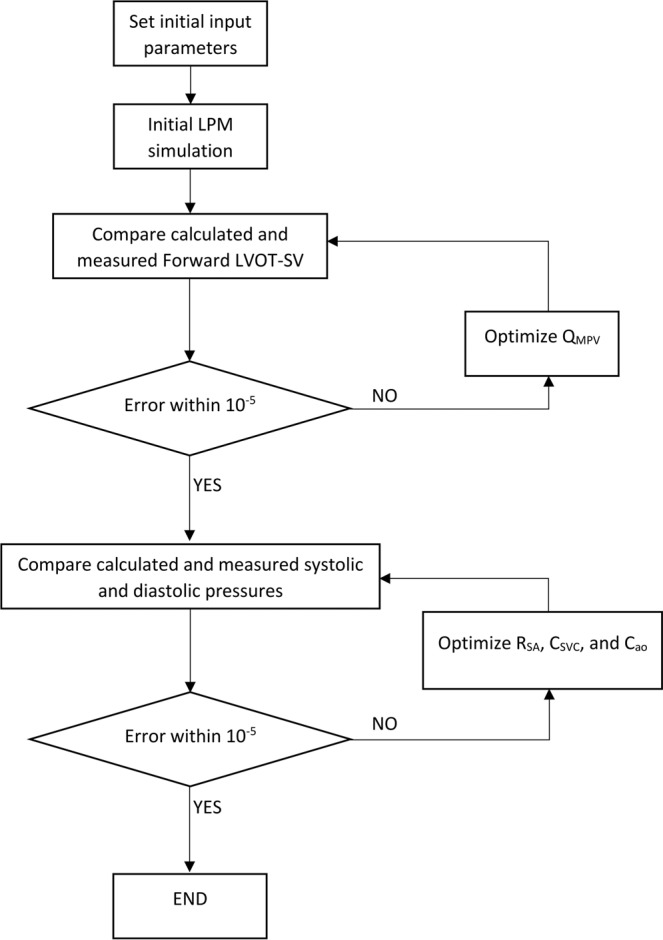
Patient-specific response optimization flow chart.

Simulink Design Optimization toolbox was used to optimize the response of the lumped-parameter model using the trust region reflective algorithm implemented in Matlab fmincon function. The response optimization was performed in two sequential steps with tolerances of 10^−6^ (Fig. 8, flow chart). In the first step, Q_MPV_, the mean flow rate of the pulmonary valve, was optimized to minimize the error between the *Forward LVOT-SV* calculated by the lumped-parameter model and the one measured in each patient. In the second step, R_SA_, C_SVC_, and C_ao_ were optimized so that maximum and minimum of the aorta pressure were respectively equal to the systolic and diastolic pressures measured using a sphygmomanometer in each patient.

### Study population

Forty-nine patients with C3VI who underwent TAVR or mitral valvuloplasty (see Table 2 for patients characteristics) between 2011 and 2018 at St. Joseph’s Healthcare and Hamilton Health Sciences (Hamilton, ON, Canada) and Hospital Universitario Marques de Valdecilla (IDIVAL, Santander, Spain) were retrospectively considered^6^. The protocols were reviewed and approved by the Institutional Review Boards of each institution as follows: the Hamilton Integrated Research Ethics Board (HiREB) of Hamilton Health Sciences and St. Joseph’s Healthcare, both affiliated to McMaster University and Comité de ética de la investigación con medicamentos de Cantabria of the Hospital Universitario Marques de Valdecilla. Informed consents were obtained from all human participants. All methods and measurements were performed in accordance with the relevant guidelines and regulations including guidelines of the American College of Cardiology and American Heart Association. Doppler echocardiography data were acquired at 2 time points: pre-procedure and 90-day post procedure. The model takes the following echocardiography parameters in patients as inputs: forward left ventricular outflow tract stroke volume (*Forward LVOT-SV*), cardiac cycle time (T), ejection time (T_EJ_), EOA_AV_, EOA_MV_, A_AO_, A_LVOT_, EOA_AR_, EOA_MR_. The model also uses the brachial systolic and diastolic pressures measured by sphygmomanometer. Cardiac catheterizations were performed pre intervention. The pressure gradients computed using the algorithm were compared and validated against cardiac catheterization measurements in forty-nine patients with C3VI.

### Statistical analysis

All results were expressed as mean ± standard deviations (SD). Statistical analyses were performed using SigmaStat software (Version 3.1, Systat Software, SanJose, CA, USA). Normal distribution was assessed with the Shapiro-Wilk test.

## Results

### Validation: *C3VI-CMF* results *vs. in vivo* measurements

Our novel non-invasive image-based computational mechanics tool (C3VI-CMF), described above, was validated against cardiac catheterization in 49 human subjects as follows:

#### Pressure waveforms

**Figure 9:**
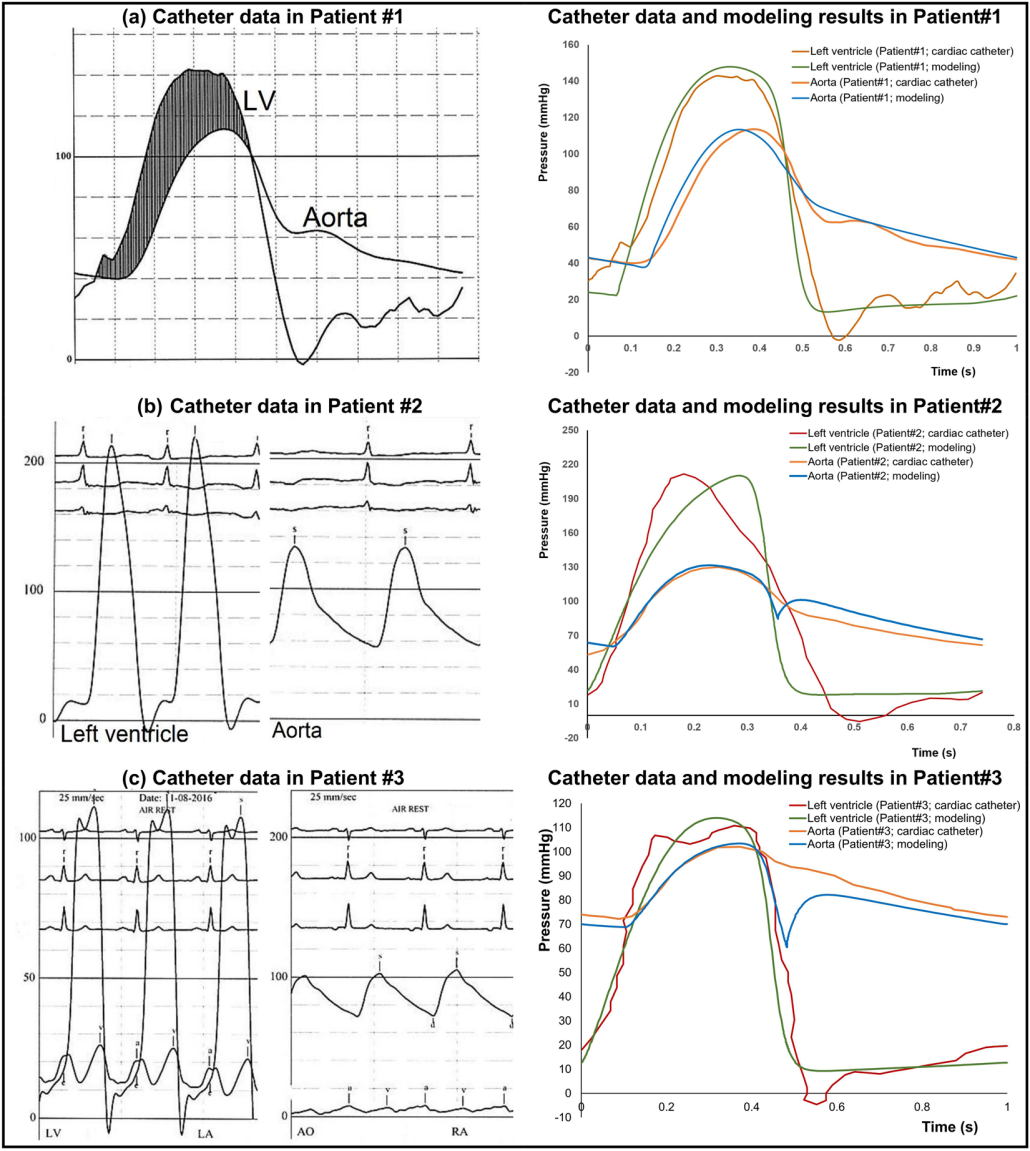
Pressure waveform comparison. Catheter data and pressure calculated by C3VI-CMF in patients with C3VI. The beat-to-beat C3VI-CMF pressure calculation compared favorably with cardiac catheter pressure measurement in all subjects.

The beat-to-beat pressure calculations of C3VI-CMF were compared with cardiac catheter pressure measurements in all 49 subjects. Figure 9 shows examples of comparisons of C3VI-CMF calculations with catheter data in 3 patients (Patients #1, #2 and #3). Results of C3VI-CMF show good qualitative agreements with catheter measurements in terms of both shape of the waveform, and specific wave features such as the amplitude and the timing of the systolic peak in the left ventricle and aorta. In all subjects (n = 49), the calculations done by C3VI-CMF had an average RMS error of 11.8 mmHg in the LV pressure, and an average RMS error of 9.9 mmHg in the aorta pressure.

#### Peak pressure

**Figure 10:**
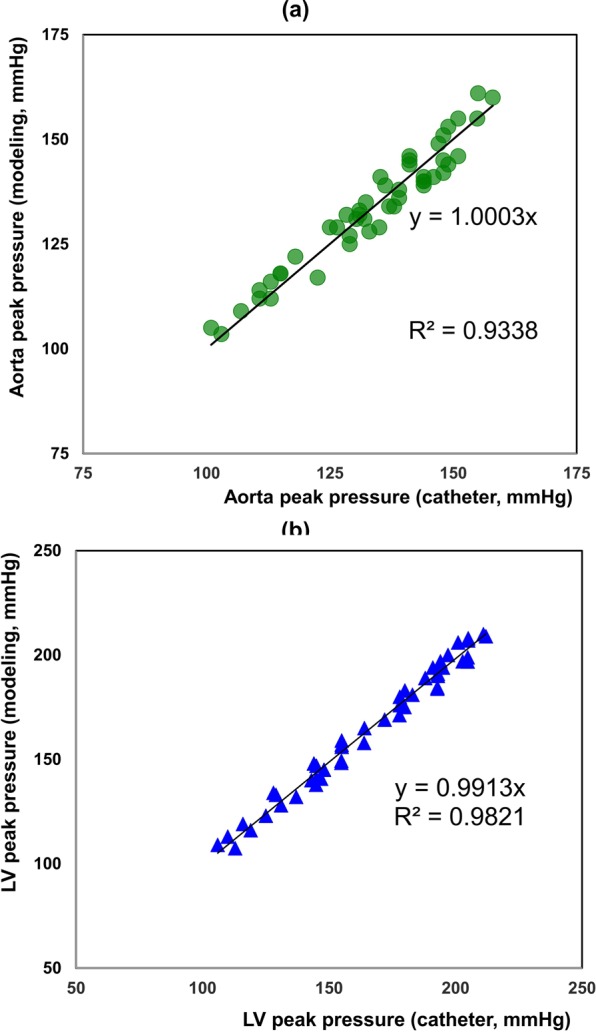
Peak pressure correlation. Peak pressures calculated by C3VI-CMF correlated well with catheter measurements in all 49 patients with C3VI as indicated by high coefficients of determination. (**a**) Left ventricle; (**b**) Aorta.

The Peak pressures calculated by C3VI-CMF (LV: 164.5 ± 30.7 mmHg, aorta: 133.88 ± 14.25 mmHg) were in close agreement with the catheter measurements (LV: 165.9 ± 30.9 mmHg, aorta: 133.75 ± 14.67 mmHg) in all subjects (n = 49). Peak pressures resulted from C3VI-CMF correlated well with the catheter measurements as indicated by high coefficients of determination in Fig. 10 (LV: R^2^ = 0.982; aorta: R^2^ = 0.933). Maximum relative errors of 4.49% and 4.33% were respectively observed in the aorta and LV pressure in all C3VI subjects, consistent with high correlations.

### *C3VI-CMF* quantifies hemodynamics metrics of circulatory and cardiac function

#### Metrics of circulatory function

The sophisticated vascular network connected to the heart, impose boundary conditions on it. As the local flow dynamics are influenced by downstream and upstream conditions, replicating correct flow and pressure conditions is critical in developing a patient-specific cardiovascular simulator. This not only gives patient-specific flow and pressure conditions to the local flow but also enables investigation of the effects of local hemodynamics on the global circulatory physiology. Investigating the details of flow and pressures in the presence of C3VI is very challenging because of the interactions between disease constituents and amplifying adverse effects of one another. Although cardiac catheterization is the gold standard for evaluating pressure and flow through the heart and circulatory system in clinics, it is invasive, expensive, and high risk and therefore not practical for diagnosis in routine daily clinical practice or serial follow-up examinations. Most importantly, cardiac catheterization only provides access to the blood pressure in very limited regions rather than details of the physiological pulsatile flow and pressures throughout the heart and the circulatory system.

**Figure 11:**
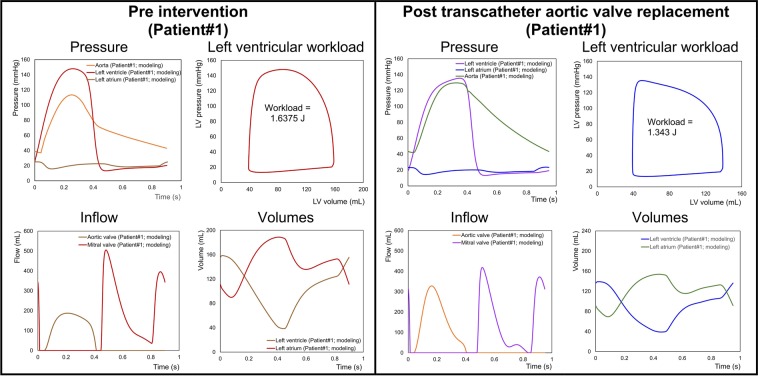
Example of predicted hemodynamics in a C3VI patient (Sample case#1) from baseline to 90 days post-TAVR. *Pre-TAVR:* severe aortic stenosis (EOA = 0.5 cm^2^), mild aortic regurgitation (AR), moderate to severe mitral regurgitation (MR), moderate to severe concentric hypertrophy, ejection fraction: 50%, brachial pressures: 40 and 115 mmHg, forward LV stroke volume: 54 mL; *Post-TAVR*: aortic valve (EOA = 1.6 cm^2^), mild to moderate paravalvular leakage, moderate to severe MR, hypertension, moderate to severe concentric hypertrophy, ejection fraction: 60%, brachial pressures: 45 and 140 mmHg, forward LV stroke volume: 53 mL.

**Figure 12:**
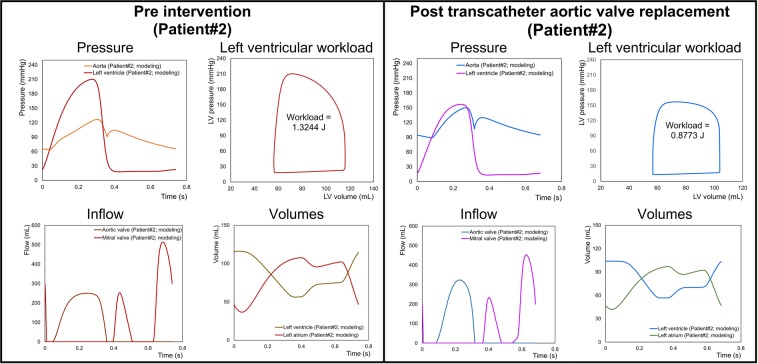
Example of predicted hemodynamics in a C3VI patient (Sample case#2) from baseline to 90 days post-TAVR. *Pre-TAVR:* severe aortic stenosis (EOA = 0.55 cm^2^), mild aortic regurgitation (AR), mild mitral regurgitation (MR), severe concentric hypertrophy, ejection fraction: 60–65%, brachial pressures: 50 and 135 mmHg, forward LV stroke volume: 52 mL; *Post-TAVR*: aortic valve (EOA = 1.45 cm^2^), trace MR, hypertension, severe concentric hypertrophy, ejection fraction: 60%, brachial pressures: 90 and 150 mmHg, forward LV stroke volume: 46 mL.

**Figure 13:**
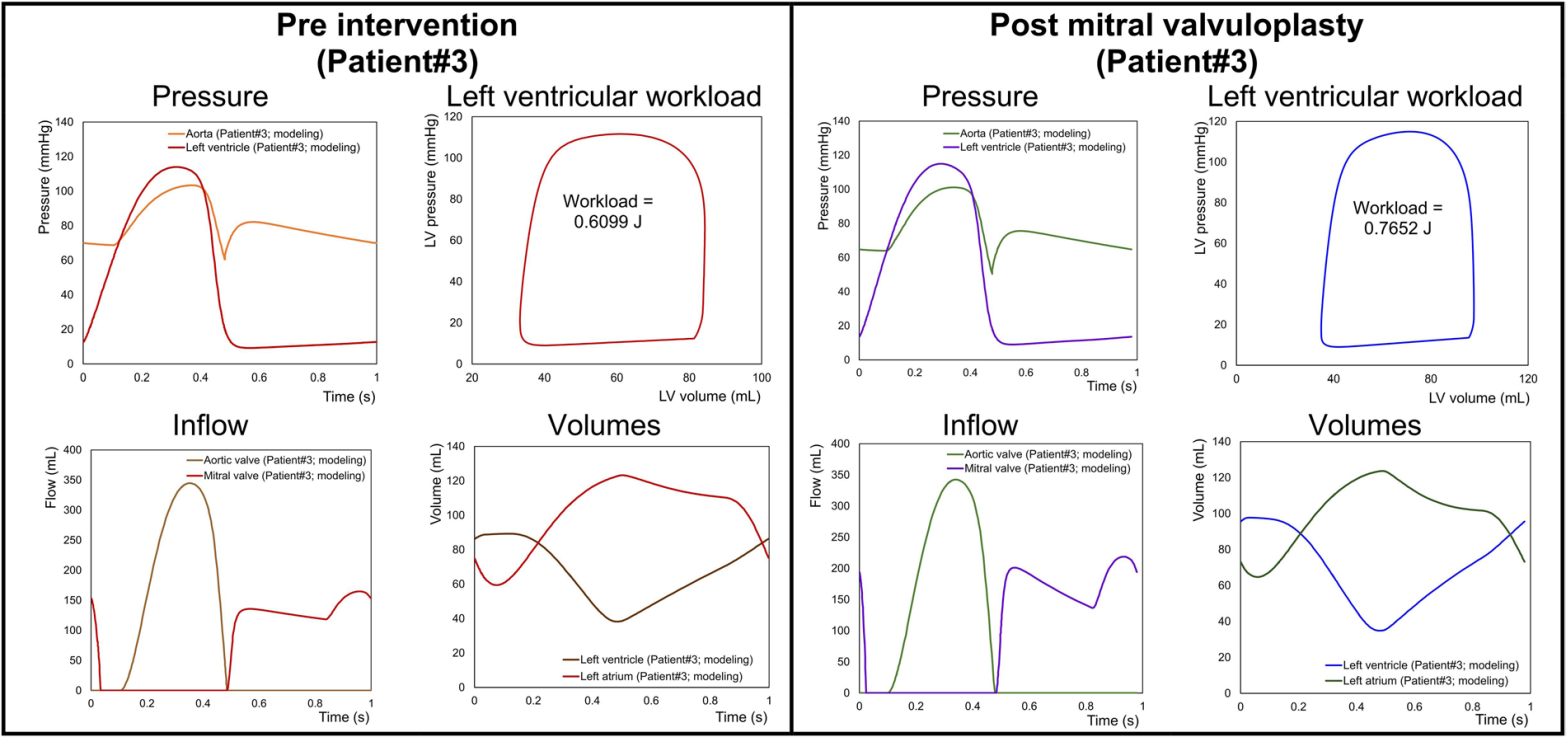
Example of predicted hemodynamics in a C3VI patient (Sample case#3) from baseline to 80 days post-valvuloplasty. *Pre-valvuloplasty*: mitral valve stenosis (EOA = 1 cm^2^), No MR, moderate AS (EOA = 1.5 cm^2^), mild AR (REOA = 0.05 cm^2^), ejection fraction: 55–60%, forward LV stroke volume: 46 mL, and brachial pressures: 70 and 105 mmHg; *Post-valvuloplasty:* mitral valve stenosis (EOA = 1.5 cm^2^), mild to moderate MR (REOA = 0.1 cm^2^), moderate AS (EOA = 1.5 cm^2^), mild AR (REOA = 0.05 cm^2^), ejection fraction: 55–60%, forward LV stroke volume: 48 mL, and brachial pressures: 62 and 100 mmHg.

In contrast, C3VI-CMF can non-invasively quantify details of the physiological pulsatile flow and pressures throughout the heart and the circulatory system in patients with C3VI. It provides instantaneous quantities such as left-ventricle pressure, aorta pressure, mitral and left-ventricle flow, left ventricle and left atrium volumes, etc. Figures 11 to 13 show samples of C3VI-CMF calculations for the same C3VI patients (Patients #1, #2 and #3) whose catheter and C3VI-CMF data for validation were shown (Fig. 9) and discussed above. Patient #1 (Fig. 11) underwent TAVR (Edwards biological prosthesis) andhad the following conditions: *Pre-TAVR*: severe calcific aortic stenosis, mild aortic regurgitation (AR), moderate to severe mitral regurgitation (MR) and moderate to severe concentric hypertrophy; *Post-TAVR*: mild to moderate paravalvular leakage, moderate to severe MR with moderate concentric hypertrophy and hypertension. Patient #2 (Fig. 12) underwent TAVR (Edwards biological prosthesis) and had the following conditions: *Pre-TAVR*: severe aortic stenosis, mild AR, mild MR and severe concentric hypertrophy; *Post-TAVR*: trace MR, moderate concentric hypertrophy and hypertension. Patient #3 (Fig. 13) underwent mitral dilatation (valvuloplasty) and had the following conditions: *Pre-valvuloplasty*: mitral valve stenosis, moderate AS and mild AR. *Post-valvuloplasty*: mitral valve stenosis, mild to moderate MR, moderate AS and mild AR. Figures 11 to 13 demonstrate that in all three patients with various C3VI disease combinations, C3VI-CMF was able to quantify details of the physiological pulsatile flow and pressures through the heart and circulatory system (local hemodynamics).

#### Metrics of cardiac function

In the presence of C3VI, the heart is overloaded since the healthy instantaneous LV pressure and/or flow are altered. There are no methods that can invasively or non-invasively quantify the heart workload (global function) and provide contribution breakdown of each component of the cardiovascular system. The heart workload is the integral of LV pressure and its volume change and was estimated as the area covered by the LV pressure–volume loop. This is especially crucial in C3VI because quantifications of the LV workload and its breakdown are vital to guide prioritizing interventions.

Figures 11 and 12 show the pre and post intervention LV workload in C3VI Patients #1& #2 who received TAVR. Pre intervention, untreated aortic stenosis increased the burden on the LV due to the augmented flow resistance which causes a LV pressure overload in the pre-intervention status. Post intervention, TAVR was accompanied by reduction in LV workload in both patients reducing the LV workload (by 27% and 33.7% in Patient #1 and #2, respectively). Figure 13 shows LV workload in Patient #3 in pre and post valvuloplasty status. Instead of improving the heart condition by reducing the LV workload, valvuloplasty caused an increase in the LV workload due to worsening the mitral regurgitation. Figures 11 to 13 demonstrate that in all three patients with various C3VI disease combinations, *C3VI-CMF* was able to quantify the heart workload (global hemodynamics).

**Figure 14:**
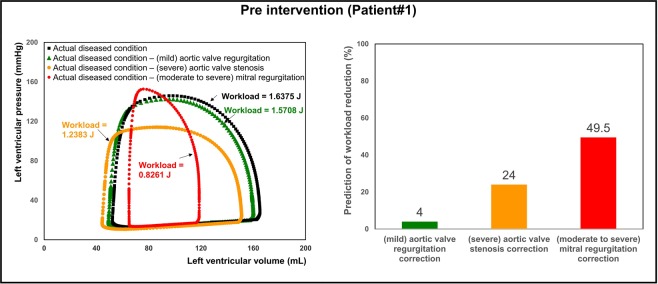
Example of workload breakdown analysis and prediction for effects of interventions in Patient #1. Right: P-V diagram of the actual diseased condition and prediction of several valve interventions. Left: Predicted percent decrease in the left ventricle workload following valve interventions. In order to plan valve interventions, each of the valvular disease constituents were replaced by the normal condition one-at-a-time and the LV workload was calculated and shown in the left panel. Both mitral valve regurgitation (49.5% increase) and aortic valve stenosis (24% increase) had substantial contributions to increasing the workload. According to this analysis, correcting of mitral valve regurgitation should have the highest priority in this patient.

Figure 14 summarizes an example of calculations for analyzing the breakdown of the contributions of the disease constituents on the LV workload in Patient #1. In the pre-intervention state, this patient had severe calcific aortic stenosis, mild aortic regurgitation, moderate to severe mitral regurgitation and concentric hypertrophy. In order to plan valve interventions, each of the valvular disease constituents were replaced by the normal condition one-at-a-time and the LV workload was calculated and shown in the left panel of Fig. 14. As the right panel of Fig. 14 shows, both mitral valve regurgitation (49.5% increase) and aortic valve stenosis (24% increase) had substantial contributions to increasing the workload. However, because mitral valve regurgitation had the greatest contribution, correcting it should have had the highest priority in the sequence of interventions. Considering the conditions of this patient, the decision of whether to also perform mitral intervention at the time of aortic valve intervention might have been carefully evaluated and considered. However, in reality, this patient only underwent transcatheter aortic valve replacement, TAVR (Fig. 11). The presented simulation results (Fig. 14) predict that fixing aortic valve stenosis alone can reduce the workloadby 24% which agrees with the actual measurement data post-intervention (Fig. 11) in this patient (workload was reduced by18% after TAVR).

## Discussions

Due to the wide inter-subject variability in cardiovascular anatomy and pathophysiology, it is ideally necessary to design individualized treatment plans based on the diagnosis data and the predictions made about individuals’ risk of the intervention. The C3VI-CMF framework developed here is an innovative patient-specific non-invasive diagnostic, monitoring, and predictive tool that can investigate and quantify effects of C3VI constituents on the heart function, and the circulatory system. The basis of C3VI-CMF is calculations of the local hemodynamics (detailed information of the fluid dynamics of the circulatory system, e.g., flow and pressure in different regions) and global hemodynamics (the heart workload). This tool can provide the breakdown of the effects of disease constituents on the global function of the heart as well so it can help predicting the effects of interventions and planning for the sequence of interventions. C3VI-CMF is capable of tracking cardiac and vascular state based on accurate time-varying models that reproduce physiological responses. While such information is vitally needed for effectively using advanced therapies to improve clinical outcomes and guiding interventions in C3VI patients, they are not currently accessible in clinic.

We evaluated our method under pathophysiologic conditions and assessed its performance in forty-nine C3VI patients with a substantial inter- and intra-patient variability with a wide range of disease. The presented results demonstrate not only repeatability but also validity even in vastly different physiologic conditions (Figs. 9 and 10; Table 2). This demonstrates the ability of C3VI-CMF to track changes in both cardiac, and vascular states. C3VI-CMF *purposefully uses reliable non-invasive input parameters to continuously calculate patient-specific hemodynamics quantities to be used for diagnosis, monitoring, and prediction of cardiac function and circulatory state with direct clinical relevance*.

C3VI-CMF can be potentially used as: (1) a personal wearable device or as a mobile application for patient monitoring; (2) a module incorporated in the software of Doppler echocardiography machines for diagnosis and prediction; and (3) a monitoring and diagnostic device for ambulatory care and intensive and critical care unit.

## Limitations

This study was performed on 49 patients with C3VI. Future studies must consider further validation of C3VI-CMF in a larger population of C3VI patients.

## Data Availability

The development and validation of the proposed method require the retrospective clinical data routinely measured in clinics (Doppler ultrasound and catheter data). These data were transferred as the de-identified & anonymized data from St. Joseph’s Healthcare and Hamilton Health Sciences (Hamilton, ON, Canada) and Hospital Universitario Marques de Valdecilla (IDIVAL, Santander, Spain)^6^. The code and the optimization algorithm used for *C3VI-CMF* are available from the author upon request.
